# Burden of disease resulting from chronic mountain sickness among young Chinese male immigrants in Tibet

**DOI:** 10.1186/1471-2458-12-401

**Published:** 2012-06-06

**Authors:** Tao Pei, Xiaoxiao Li, Fasheng Tao, Haotong Xu, Haiyan You, Linlin Zhou, Yan Liu, Yuqi Gao

**Affiliations:** 1 Department of Health Service, College of High Altitude Military Medicine, Third Military Medical University, 30 Gaotanyan Street, Shapingba District, Chongqing, 400038, P. R. China; 2 Beijing Long March Scientific and Technical Information Institute, Beijing, 100076, P. R. China; 3 Key Laboratory of High Altitude Medicine, Ministry of Education, Third Military Medical University, Chongqing, 400038, P. R. China; 4 Key Laboratory of High Altitude Medicine, PLA, Chongqing, 400038, P. R. China; 5 Department of Health Service, Urumqi General Hospital of Lanzhou Military District, Urumqi, Xinjiang, 830000, P. R. China; 6 Department of Radiology, Nanchong Central Hospital of North Sichuan Medical College, Nanchong, Sichuan, 637000, P. R. China; 7 Department of Health Service, Ngari Military Subdistrict, Tibet, 859000, P. R. China

**Keywords:** chronic mountain sickness, burden of disease, disability-adjusted life years, disability weight, immigrant, high altitude

## Abstract

**Background:**

In young Chinese men of the highland immigrant population, chronic mountain sickness (CMS) is a major public health problem. The aim of this study was to measure the disease burden of CMS in this population.

**Methods:**

We used disability-adjusted life years (DALYs) to estimate the disease burden of CMS. Disability weights were derived using the person trade-off methodology. CMS diagnoses, symptom severity, and individual characteristics were obtained from surveys collected in Tibet in 2009 and 2010. The DALYs of individual patients and the DALYs/1,000 were calculated.

**Results:**

Disability weights were obtained for 21 CMS health stages. The results of the analyses of the two surveys were consistent with each other. At different altitudes, the CMS rates ranged from 2.1-37.4%; the individual DALYs of patients ranged from 0.13-0.33, and the DALYs/1,000 ranged from 3.60-52.78. The age, highland service years, blood pressure, heart rate, smoking rate, and proportion of the sample working in engineering or construction were significantly higher in the CMS group than in the non-CMS group (*p* < 0.05). These variables were also positively associated with the individual DALYs (*p* < 0.05). Among the symptoms, headaches caused the largest proportion of DALYs.

**Conclusion:**

The results show that CMS imposes a considerable burden on Chinese immigrants to Tibet. Immigrants with characteristics such as a higher residential altitude, more advanced age, longer highland service years, being a smoker, and working in engineering or construction were more likely to develop CMS and to increase the disease burden. Higher blood pressure and heart rate as a result of CMS were also positively associated with the disease burden. The authorities should pay attention to the highland disease burden and support the development and application of DALYs studies of CMS and other highland diseases.

## Background

Globally, more than 140 million people worldwide live > 2500 m above sea level; of them, 80 million live in Asia, and 35 million live in the Andean mountains [1]. Countless others travel to the mountains for work, trade, and sport. China has the largest and highest highland territory in the world, and there is a large immigrant population in this territory [2]. The immigrant population is composed mainly of road builders, miners, servicemen, officials, and merchants, most of whom are adult men. The ethnicity of the population is mostly Han (ethnic Chinese) [2,3]. Han are at a greater disadvantage than their Tibetan counterparts regarding adaptation to high-altitude environments [4,5].

Despite a long acclimatization period, some immigrants in the highlands suffer from chronic mountain sickness (CMS), which is also called Monge disease [6]. CMS is a hypobaric, hypoxia-related illness that presents with polycythemia leading to cardiac failure or neurological disorders [7]. CMS seriously affects the health of highland immigrants and often results in significant declines in productivity and quality of life. Patients usually experience decreased exercise tolerance, loss of memory, headache, dizziness, and fatigue [8,9]. Compared with their Tibetan counterparts, Han suffer from significantly higher rates of CMS when they reside in highland areas [1,10,11].

However, the decline in health status induced by CMS in the immigrant population has been underestimated in the past. The authorities focus on acute mountain sickness because it can cause obvious loss of manpower in some emergency situations. By contrast, CMS, of which the symptoms are generally not severe, tends to be ignored by the authorities. They believe that CMS does not affect day-time work very much, and it can be overcome by will power and courage derived from military discipline and patriotism. However, the disease not only gradually reduces the state of health and quality of life of individuals, but it also decreases the working capacity of the whole population. In the health service system for Chinese highland troops, CMS has not been considered to be an occupational disease or to warrant compensation. Currently, the highland troops only calculate the prevalence of CMS. Quality of life is not considered to be important by the authorities, and the reduction in health cannot be quantitated. Consequently, health service plans and occupational compensation for CMS cannot be assessed at the population level by calculating CMS prevalence alone. The health-related quality of life of different populations may be unequal because the severity of CMS has not been considered. Even when the CMS severity is the same, old and young patients differ in the extent to which they experience a loss of productivity. At the individual level, although a scoring system may reveal the severity of CMS, it fails to quantitatively evaluate the health life years of which a patient is deprived by CMS; the cumulative damage of CMS to a person’s health cannot be determined. Consequently, health services and prevention strategies cannot be conducted effectively, and working standards and disability compensation cannot be set rationally. Therefore, it is necessary to evaluate the disease burden of CMS to improve such policies.

Disability-adjusted life years (DALYs) have been employed to quantify the burden of diseases, taking into account healthy years of life lost due to premature death, as well as ill-health [12]. The concept of DALYs was developed as a measurement unit to quantify burden of disease (BOD) and injury to human populations for the global burden of disease (GBD) study in the early 1990s [13,14]. Since then, DALYs have been widely used globally to estimate the BOD at the national, international, and regional levels [15]. The DALYs approach represents a major step in quantifying the global and regional effects of diseases on population health, and it provides insights for organizations and individuals who are committed to health policy [16]. The concept of BOD can provide important insights for high-altitude medical research. However, until now, little attention has been paid to the BOD of high-altitude populations. Accordingly, this study aimed to establish the disability weights (DWs) of CMS and to estimate the burden of CMS among young Chinese men in Tibet.

## Methods

### Ethics statement

This study was approved by the Ethics Review Board of the Third Military Medical University, Chongqing, China. There was no health intervention involving the subjects. All the individual data were anonymized prior to retrieval and analysis. Prior to commencement of the study, all the subjects were fully informed and signed an informed consent document.

### Selection of population groups

The areas surrounding Qinghai-Tibet Road (1,138 km) and Xinjiang-Tibet Road (1,530 km) were selected as the study areas. These roads are both stem roads in Tibet, and most military units in the area have conditions that are considered to be representative. Every year, the Highland Medical Corps conducts compulsory, routine health surveys of their troops to determine the condition of the servicemen’s health. From April to June 2009 and from April to June 2010, we accompanied the Corps to conduct the present study. We randomly selected 41 units (one fourth of the units completing routine health surveys) for this study. The altitudes of these units ranged from 3,570-5,380 m, with an average of 4,350.9 m (standard deviation, SD = 424.2). We classified these altitudes into four groups: 3,500-3,999 m (8 units), 4,000-4,499 m (15 units), 4,500-4,999 m (14 units), and 5,000-5,400 m (3 units). The 2009 and 2010 surveys were conducted in the same area and with the same units.

### Data collection

During data collection, we accompanied the Medical Corps and visited the sample units. The subject selection criteria included the following: 1) ethnic Han; 2) having resided in the highland for more than six months; 3) coming from a low-altitude province (< 2,500 m); 4) without a current, acute infection; 5) without any chronic pulmonary diseases or any other severe chronic diseases (e.g. skin diseases, oral diseases, digestive diseases, musculoskeletal disorders, and soft tissue injury).

In each sample unit, the Medical Corps first performed their routine survey in each unit’s clinic room, which included a physical examination, behavioral questions, and a blood draw (from an arm vein). We extracted information about height (in meters), weight (in kilograms), blood pressure (BP, in mmHg), heart rate (HR, in beats/min), and current smoking and drinking behavior (current Yes or No) from the routine survey. We also extracted the patient’s age (in years), highland service years (HSYs), educational level (classified as ≤ elementary school, high school, or ≥ college), and occupation (classified as regular infantry, engineering or construction, or support) from the units’ staff records.

Accompanying the routine examination, we obtained each patient’s oxygen saturation (SaO_2_) and hemoglobin concentration (Hb). The SaO_2_ was tested using a pulse oximeter (GE Datex-Ohmeda TuffSat, USA) on the index finger. Hb was measured using the cyanmethemoglobin technique (Hb assay kit, MAKER, Sichuan, China; Hb 1002 analyzer, Shanghai Scientific Instrument Corporation, Shanghai, China).

The CMS diagnostic criteria included excessive hypercythemia (Hb ≥ 210 g/L) and low oxygen saturation (SaO_2_ < 85%) [17,18]. Subjects who were diagnosed with CMS completed a symptom questionnaire. The CMS symptom questionnaire was based on the Qinghai CMS Questionnaire [18], which includes questions about seven main CMS symptoms (breathlessness and/or palpitations, sleep disturbances, cyanosis, dilatation of veins, paresthesia, headache, and tinnitus). The participants were asked to rate each symptom according to three severity levels (mild, moderate, and severe) during the past month. During the questionnaire, they were not informed of the diagnosis. After the completion of the questionnaire, all the CMS subjects’ answers were thoroughly reviewed to check for missing values. All missing values were completed before the observers left the unit.

Subjects who were not diagnosed with CMS did not undergo any further testing; however, we randomly selected a group of non-CMS subjects that was twice the size of the CMS group to be a control group for each unit’s data in the data analysis. Any non-CMS subjects with missing values were excluded from the study, and the data were complemented by another non-CMS subject in the same unit.

### Disability weights for CMS

Disability weight (DW) is a key component of BOD analysis that represents the severity of an illness. DW ranges from 0 to 1, where 0 represents healthy life and 1 represents death [19]. The GBD study group derived a series of DWs for different health states that are the outcomes of different diseases [20]. However, no DW is available for a number of health states, including CMS. There are also no DWs of other diseases that are comparable to the health states associated with CMS in the 2004 report of the GBD study group [20]. Because of the non-availability of DWs for CMS, we tried to establish our own DWs for CMS health states by using the Qinghai CMS Questionnaire symptom categories. There were seven symptoms, and each symptom included 3 levels of severity (mild, moderate, and severe), thus representing 21 CMS health states.

We convened a nine-expert panel composed of 3 physiologists, 2 epidemiologists, 3 public health experts, and 1 highland medical officer. To create the DWs, the panel members performed the Person Trade-Off (PTO) exercise using a Delphi process [21]. In the beginning, they were asked to decide how many people with a given CMS health state would need to be returned to perfect health for one year to provide the same social benefit as 1,000 healthy people living for one year (PTO value). During the first round, each expert provided PTO values for each of the 21 CMS health states. The panel then met as a group and discussed each CMS health state in turn. At the start of the discussion, all the panelists received the first round’s PTO values, provided anonymously by each of the panelists. The group discussed the most appropriate PTO value for each CMS state. During the discussion all opinions were respected. When distinctive thematic considerations were no longer elicited, each panelist was asked to independently and confidentially reassign a PTO value to each CMS state. The coefficient of variation (CV) was calculated to determine the need for additional rounds of discussion and reassignment of values. After 4 rounds, a consensus was achieved (CV < 0.5). In all, 5 items required only 1 round, 12 items required 2 rounds, and 4 items required 3 rounds, at which point the Delphi process ended. The PTO values were converted to DWs with 95% confidence intervals (95% CI).

To describe the health states, this study used a standardized, eleven-dimension description (pain or discomfort, physical functioning, fatigue, memory and thinking, social relationships, anxiety, speech, hearing, vision, and the use of hands and fingers) of the associated functional health states for each disease stage [22,23]. Each dimension had three levels: no problems, some problems, and severe problems. The health states were described on an A4-sized vignette that contained disease-specific information in simple terminology. As a reference framework during this task, the panel members were provided with a WHO-GBD framework table, which displayed 7 disability classes and 22 anchoring example conditions [24].

### Calculation of DALYs for CMS

DALYs were used to estimate the disease burden due to CMS among young male immigrants. The DALYs for a disease or health condition are calculated as the sum of the year of life lost (YLL) due to premature mortality in the population and the equivalent ‘healthy’ year lost due to disability (YLD) for incident cases of the health condition [25]. However, people with CMS normally do not die directly from the disease, especially in a young population, nor was mortality due to CMS reported during the 2009–2010 CMS epidemics in the region. Therefore, the DALY due to CMS were equal to the YLD.

Disease stages were evaluated by assuming a duration of 1 year, except for those with only a brief duration and those followed by a full recovery [26]. Given that CMS is a non-communicable chronic disease; in most cases, remission appears when the patient is returned to a lower area, accordingly, we assumed that the duration of one subject’s CMS symptoms was an entire year and that the severity of the CMS symptoms was averaged across the year. We obtained the symptoms of each patient with CMS during a cross-sectional investigation performed each year, and used the time-section-symptoms to represent the patients’ symptoms for the whole year. A patient’s individual DALYs were calculated case-by-case using the following formula [27]:

(1) $$YLDs[r,K,\beta ]=D\frac{KCe^{\mathrm{ra}}}{{\left(r+\beta \right)}^{2}}\left\{\left[e^{-(r+\beta )(L+a)}\left[-(r+\beta )(L+a)-1\right]-e^{-(r+\beta )a}\left[-(r+\beta )a-1\right]\right]+\frac{1-K}{r}(1-e^{-rL})\right\}$$

In this formula, *D* is DW, *K* is an age weighting factor, *C* is a constant, *r* is the discount rate, *a* is age at beginning, *β* is a parameter from the age weighting function, and *L* is life time with disability. We used the base case recommended by Murray and Lopez, with *C* = 0.1658, *r* = 0.03, *K* = 1, and *β* = 0.04 [28]. As far as comorbidity was concerned, if a person had more than one CMS symptom, a multiplicative adjustment method was adopted to calculate the DW of the comorbidity [29]. The adjustment formula is:

(2) $$w_{\left(d\right)}=1-\prod_{d}(1-w_{d})$$

Where W_(d)_ is the total DW of an individual with multiple (d) symptoms, and w_d_ is the DW of each health state. For example, if one person complained of both “poor night’s sleep” (DW = 0.113) and “palpitations” (DW = 0.129), his total DW would be calculated as 1–(1–0.113) × (1–0.129) = 0.227.

To estimate the differences in disease burden of CMS in different groups (e.g., groups at different altitudes), we calculated the average disease burden in each group as the DALYs per 1,000 people (DALYs/1,000).

### Statistical analysis

To control for potential changes in CMS status across the two years of the study, the two data subsets were analyzed separately.

Descriptive statistics were calculated for the age, body mass index (BMI, weight in kg/height in m^2^), HSYs, systolic blood pressure (SBP), diastolic blood pressure (DBP), HR, SaO_2_, Hb, smoking, drinking, and occupation of the CMS and non-CMS subjects. The CMS prevalence, individual DALYs, and DALYs/1,000 in each altitude group were computed. The proportions of disease burden caused by each symptom in each year in the entire sample population were also computed. Differences between the means were tested by an analysis of variance (ANOVA), and differences in rates or proportions were tested with a chi-squared test.

To test the relationship between the individual DALYs and the individual variables of age, BMI, HSYs, SBP, DBP, HR, smoking, drinking, and occupation, separate linear regression analyses were performed using the individual DALYs as dependent variables and each individual-level variable as the independent variable, while controlling for altitude. Variables that were significant in each of the separate regressions were entered into a multiple regression model. Categorical variables were transformed into dummy variables for the analysis. Because Hb and SaO_2_ are diagnostic factors, they were not entered in regression analysis to avoid a vicious circle. The standardized regression coefficients (*β*) with 95% CI and the *p*-values from the analyses are reported.

Before all tests, the normality and homogeneity of variance were tested for each continuous variable. A log transformation was used for variables with non-normality or non-homogeneity of variance. In all the analyses, a *p-*value <0.05 was considered to be statistically significant. SPSS 13.0 software was used (Chicago, IL, USA).

## Results

### Disability weights of CMS

After three rounds of the Delphi process, the panel reached a consensus with a CV < 0.5. In this way, the DWs of 21 CMS health stages were obtained. Severe headache received the highest weight of 0.231, while mild cyanosis received the lowest weight of 0.006, as shown in Table 1. The fourth and fifth columns in the table show the upper and lower 95% CI of the DWs, and the sixth column shows the CVs from when the panel achieved a consensus.

**Table 1:** Disability weights for the 21 health states from the 7 symptom categories of chronic mountain sickness

**Symptom categories**	**Health stages**	**DWs**	**95% CI**		**CV**
			**Lower**	**Upper**	
**Breathlessness/palpitations**	Mild breathlessness/palpitations	0.049	0.044	0.053	0.15
	Moderate breathlessness/palpitations	0.091	0.086	0.096	0.09
	Severe breathlessness/palpitations	0.129	0.120	0.139	0.11
**Sleep disturbances**	Did not sleep as well as usual	0.043	0.040	0.046	0.09
	Woke many times; poor night’s sleep	0.113	0.108	0.117	0.06
	Could not sleep at all	0.218	0.207	0.229	0.08
**Cyanosis**	Mild cyanosis	0.006	0.005	0.007	0.36
	Moderate cyanosis	0.016	0.013	0.020	0.33
	Severe cyanosis	0.040	0.035	0.046	0.19
**Dilatation of veins**	Mild dilatation of veins	0.009	0.007	0.011	0.32
	Moderate dilatation of veins	0.021	0.015	0.027	0.42
	Severe dilatation of veins	0.044	0.036	0.052	0.28
**Paresthesia**	Mild paresthesia	0.014	0.012	0.017	0.27
	Moderate paresthesia	0.042	0.039	0.044	0.10
	Severe paresthesia	0.083	0.078	0.089	0.11
**Headache**	Mild headache symptoms	0.077	0.071	0.083	0.12
	Moderate headache	0.131	0.127	0.136	0.05
	Severe, incapacitating headache	0.231	0.224	0.238	0.05
**Tinnitus**	Mild tinnitus	0.014	0.012	0.017	0.27
	Moderate tinnitus	0.049	0.045	0.052	0.11
	Severe tinnitus	0.095	0.092	0.099	0.06

DWs: disability weights; CV: coefficient of variation; 95% CI: 95% confidence interval.

### Variables between CMS and non-CMS

The descriptive statistics for each continuous, individual variable in the CMS and non-CMS groups are shown by year in Table 2. The population of sample units differed little between the two years. In 2009, the total sample Han population was 2,277; the population of each unit ranged from 23 to 139 individuals, with an average of 55.5 (SD = 24.1). In 2010, the total sample population was 2,155; the population of each unit ranged from 21 to 150 individuals, with an average of 52.6 (SD = 25.5). Compared with the non-CMS group, the CMS group was significantly older (2009: *p* < 0.001; 2010: *p* = 0.004), had more HSYs (2009: *p* < 0.001; 2010: *p* = 0.010), and had a higher SBP (2009: *p* < 0.001; 2010: *p* < 0.001), DBP (2009: *p* = 0.002; 2010: *p* < 0.001), and HR (2009: *p* < 0.001; 2010: *p* < 0.001). BMI was not found to be significant (2009: *p* = 0.131; 2010: *p* = 0.641).

**Table 2:** Means and standard deviations of each continuous individual variable in CMS and non-CMS subjects in 2009 and 2010

**Variable**	**Mean (SD)**	
	**2009**^**†**^	**2010**^**‡**^
**Age (years)**		
CMS	23.45 (3.73)^*^	23.31 (3.57)^*^
Non-CMS	22.51 (2.95)	22.61 (3.46)
**Body mass index (kg/m**^**2**^**)**		
CMS	21.69 (2.56)	21.56 (2.78)
Non-CMS	21.42 (2.65)	21.48 (2.51)
**Highland service years**		
CMS	4.24 (3.33)^*^	4.10 (3.24)^*^
Non-CMS	3.44 (2.66)	3.53 (2.98)
**Systolic blood pressure (mmHg)**		
CMS	120.98 (11.47)^*^	121.77 (10.74)^*^
Non-CMS	116.96 (10.27)	118.41 (9.45)
**Diastolic blood pressure (mmHg)**		
CMS	77.31 (11.32)^*^	78.30 (12.67)^*^
Non-CMS	75.07 (7.65)	73.80 (7.78)
**Heart rate (beat/min)**		
CMS	81.17 (10.74)^*^	80.06 (8.53)^*^
Non-CMS	76.63 (8.13)	77.07 (8.02)
**Hemoglobin (g/L)**		
CMS	216.68 (5.62)^*^	216.10 (5.74)^*^
Non-CMS	191.05 (12.69)	190.94 (13.54)
**Oxygen saturation (%)**		
CMS	82.45 (2.69)^*^	81.69 (2.41)^*^
Non-CMS	88.85 (3.43)	89.01 (3.50)

SD: standard deviation; CMS: chronic mountain sickness.

*: *p* < 0.05 vs. control based on ANOVA; †: CMS n = 321, non-CMS n = 642; ‡: CMS n = 315, non-CMS n = 630.

The descriptive statistics for each categorical variable in the CMS and non-CMS groups are presented by year in Table 3. The CMS group had a higher proportion of smokers and engineering or construction (E&C) workers. In the CMS group, 61.99-62.54% were smokers and 35.24-39.88% were E&C workers, compared with the non-CMS group, in which 47.94-54.67% were smokers and 18.85-21.59% were E&C workers. Neither educational differences nor the proportion of respondents in each group who drank was found to be significant. Finally, there was no significant difference between the regular infantry and the support categories in the rate of CMS (2009: χ^2^ = 1.013, *p* = 0.314; 2010: χ^2^ = 0.645, *p* = 0.422).

**Table 3:** Statistics for each categorical individual variable by CMS and non-CMS groups in 2009 and 2010

**Variable**			**2009**			**2010**		
	**CMS**	**Non-CMS**	p^*^	**CMS**	**Non-CMS**	p^*^	
**Smoking**						
No	122	291	0.030	118	328	<0.001
Yes	199	351		197	302	
**Drinking**						
No	287	555	0.191	277	547	0.630
Yes	34	87		38	83	
**Education**						
≤Junior school	132	268	0.261	145	260	0.338
High school	173	325		142	315	
≥College	16	49		28	55	
**Occupation**						
Regular infantry	117	294	<0.001	124	284	<0.001
Engineering and construction	128	121		111	136	
Support	76	227		80	210	
**Total**	321	642		315	630	

*: *p*-value based on chi-squared test.

### CMS prevalence and estimates of DALYs

The numbers of CMS patients, prevalence, average individual DALYs, and DALYs/1,000 in the different altitude groups are presented in Table 4. There were 321 and 315 patients in the sample populations reported in the 2009 and 2010 surveys, respectively. In 2009, the prevalence ranged from 2.1-37.4%, with an average of 14.1% in the different altitude groups. In 2010, the prevalence ranged from 3.2-33.6%, with an average of 14.8%. Using the chi-squared test, the CMS rate was found to be significantly higher in the higher altitude groups (2009: χ^2^ = 59.564, *p* < 0.001; 2010: χ^2^ = 108.306, *p* < 0.001). There were no significant differences in prevalence in different years within the same altitude group (*p* > 0.05).

**Table 4:** Annual CMS patient numbers, prevalence, average individual DALYs, and DALYs/1,000 in each altitude group in 2009 and 2010

**Altitude**			**2009**					**2010**		
	N^*^	n (%)^**^	**DALY**^**†**^	**DALY/1,000**^**‡**^	N^*^	n (%)^**^	**DALY**^**†**^	**DALY/1,000**^**‡**^	
**3,500-3,999 m**	569	12 (2.1)	0.13 (0.11)	3.60 (2.26)	534	17 (3.2)	0.17 (0.11)	4.01 (3.49)
**4,000-4,499 m**	889	123 (13.8)	0.24 (0.13)	31.80 (16.62)	896	133 (14.8)	0.23 (0.12)	35.71 (19.20)
**4,500-4,999 m**	687	137 (19.9)	0.26 (0.11)	52.24 (17.88)	603	124 (20.6)	0.26 (0.09)	52.78 (22.94)
**5,000-5,400 m**	131	49 (37.4)	0.33 (0.08)	112.21 (40.33)	122	41 (33.6)	0.30 (0.11)	110.67 (28.20)

*: total number in each altitude group; **: number of patients (with prevalence in %); †: average individual DALYs of each altitude group (with the standard deviation in brackets); ‡: DALYs per 1,000 of each altitude group (with the standard deviation in brackets).

In 2009, the average individual (CMS patient) DALYs ranged from 0.13-0.33 in the various altitude groups, with an average of 0.25 (SD = 0.12). In 2010, the DALYs ranged from 0.17-0.30, with an average of 0.25 (SD = 0.11). In 2009, the DALYs/1,000 (entire population) ranged from 3.60-52.24 in the various altitude groups, with an average of 35.54 (SD = 15.37). In 2010, it ranged from 4.01-52.78, with an average of 37.09 (SD = 16.96). The one-way ANOVA found that both the individual DALYs (2009: *F* = 7.251, *p* < 0.001; 2010: *F* = 13.979, *p* < 0.001) and the DALYs/1,000 (2009: *F* = 151.547, *p* < 0.001; 2010: *F* = 154.286, *p* < 0.001) were significantly higher in the higher altitude groups. We also found that neither the individual DALYs nor the DALYs/1,000 were significantly different between the two years at the same altitude (*p* > 0.05).

The proportion of the disease burden caused by the 7 CMS symptoms in the entire sample population is presented in Table 5. In the two annual surveys, the proportions of the disease burden caused by each symptom were similar; the disease burden caused by headaches made up the largest proportion in the population (33.5% in 2009 and 32.2% in 2010), whereas breathlessness/palpitations and sleep disturbances made up the second- and third-largest proportions, respectively.

**Table 5:** Each CMS-symptom-caused DALYs/1,000 and its proportion in the entire sample population in 2009 and 2010

**Symptoms**			**2009**		**2010**	
	**DALYs/1,000**^**†**^	**Proportion (%)**^**‡**^	**DALYs/1,000**^**†**^	**Proportion (%)**^**‡**^	
Breathlessness/palpitations	8.55	21.5	8.88	21.4
Sleep disturbances	8.71	21.9	10.71	25.8
Cyanosis	1.23	3.1	1.03	2.5
Dilatation of veins	1.47	3.7	1.57	3.8
Paresthesia	3.21	8.1	2.77	6.7
Headache	13.31	33.5	13.40	32.2
Tinnitus	3.29	8.3	3.20	7.7

†: The DALYs/1,000 for each symptom were calculated by the sum of the DALYs due to each symptom divided by the total sample population; ‡: The proportion of each symptom was calculated by the sum of the DALYs due to each symptom divided by the sum of DALYs.

Higher age, HSYs, SBP, DBP, and HR were found to be significantly and positively associated with the individual DALYs in the separate linear regression analyses (Table 6). Smoking and working in E&C occupations were both significantly and positively associated with individual DALYs. In the 2009 data, a support occupation was found to have a significant and negative association with individual DALYs compared with the regular infantry; however, there was no significant association in the 2010 data. BMI, drinking, and education were not found to be significant. Because age and HSYs were highly correlated (2009: *r* = 0.978, *p* < 0.001; 2009: *r* = 0.971, *p* < 0.001), age was excluded from the multiple model to avoid multicollinearity. When the significant variables were put into the multiple model, SBP and support occupation lost significance, while HR retained significance only in the 2009 data (Table 7).

**Table 6:** Separate linear regression analyses of the relationship between individual DALYs and individual variables after controlling for altitude

**Variable**			**2009 (n = 321)**			**2010 (n = 315)**		
	***β***	**95% CI**	***p***	***β***	**95% CI**	***p***	
**Age**	0.169	(0.061, 0.277)	0.002	0.167	(0.062, 0.272)	0.002
**Body mass index**	0.042	(−0.067, 0.151)	0.447	0.055	(−0.052, 0.163)	0.313
**Highland service years**	0.177	(0.069, 0.285)	0.001	0.157	(0.051, 0.262)	0.004
**Systolic blood pressure**	0.139	(0.033, 0.245)	0.010	0.120	(0.015, 0.226)	0.026
**Diastolic blood pressure**	0.284	(0.180, 0.388)	<0.001	0.214	(0.107, 0.321)	<0.001
**Heart rate**	0.147	(0.040, 0.253)	0.007	0.152	(0.046, 0.258)	0.005
**Smoking**						
No (ref.)	0.000			0.000		
Yes	0.173	(0.068, 0.278)	0.001	0.143	(0.038, 0.247)	0.008
**Drinking**						
No (ref.)	0.000			0.000		
Yes	−0.014	(−0.121, 0.093)	0.794	0.037	(−0.069, 0.143)	0.490
**Education**						
≤Elementary school(ref.)	0.000			0.000		
High school	−0.029	(−0.140, 0.081)	0.605	−0.101	(−0.211, 0.008)	0.069
≥College	0.047	(−0.064, 0.157)	0.406	0.053	(−0.056, 0.163)	0.340
**Occupation**						
Regular infantry (ref.)	0.000			0.000		
Engineering and construction	0.162	(0.051, 0.273)	0.004	0.159	(0.050, 0.267)	0.004
Support	−0.125	(−0.237, -0.014)	0.028	−0.108	(−0.218, 0.001)	0.052

ref.: reference; *β*: regression coefficient of each variable or category; 95% CI: 95% confidence interval of each *β*; *p*: *p*-value of each *β*.

**Table 7:** Multiple linear regression analyses of the relationship between individual DALYs and individual variables after controlling for altitude

**Variable**			**2009 (n = 321)**			**2010 (n = 315)**		
	***β***	**95% CI**	***p***	***β***	**95% CI**	***p***	
**Altitude**	0.254	(0.147, 0.360)	<0.001	0.204	(0.098, 0.310)	<0.001
**Highland service years**	0.148	(0.045, 0.251)	0.005	0.135	(0.031, 0.239)	0.011
**Systolic blood pressure**	−0.021	(−0.139, 0.098)	0.732	−0.048	(−0.170, 0.074)	0.438
**Diastolic blood pressure**	0.183	(0.061, 0.304)	0.003	0.257	(0.137, 0.376)	<0.001
**Heart rate**	0.120	(0.014, 0.225)	0.026	0.085	(−0.023, 0.193)	0.124
**Smoking**						
No (ref.)	0.000			0.000		
Yes	0.113	(0.010, 0.216)	0.031	0.136	(0.035, 0.236)	0.008
**Occupation**						
Regular infantry (ref.)	0.000			0.000		
Engineering and construction	0.143	(0.030, 0.257)	0.013	0.150	(0.036, 0.264)	0.010
Support	0.091	(−0.022, 0.205)	0.114	0.077	(−0.036, 0.191)	0.181

ref.: reference; *β*: regression coefficient of each variable or category; 95% CI: 95% confidence interval of each *β*; *p*: *p*-value of each *β*.

## Discussion

As a burden of disease specific to a particular region, this study focused on CMS in the highland immigrant population. To our knowledge, this work is the first to introduce the concept of DALYs to highland medicine. In this study, we established the local DWs for CMS according to seven main symptoms, which have not been considered in previous global or regional BOD studies. This study is also the first to estimate the burden of CMS among the young male Chinese immigrant population in Tibet and it was based on the data from two recent annual surveys.

Most BOD studies use the selection of diseases states from the International Classification of Disease, Injuries, and Causes of Death, Tenth Revision (ICD-10) [30-32]. In the ICD-10, CMS (namely, secondary polycythemia due to high altitude) is coded D75.1 [33], but there are no further subdivided consequence categories. Considering the multiple symptoms characteristic of CMS, we did not merely establish a single DW for CMS because this illness is not characterized by a single health problem. In addition, it is difficult to accurately measure the DALYs with a single DW because the condition has a variable presentation that includes several different symptoms, each with its own severity level. Thus, we derived DWs for the detailed CMS symptoms and 21 potential health states that can result from those symptoms at varying degrees of severity. This division made the DWs more detailed and easier to handle. The calculation of the DWs in this study was based on the PTO methodology and used a panel approach to elicit valuations of the different CMS health states. The panel included representatives from four types of specialties, which may reflect different specialties.

In this study, the overall disease burden caused by CMS among the young male Chinese immigrant population was 35.54-37.09 DALYs/1,000 in the sample region. Although there are no comparable DALYs data specific to CMS from previous studies, we compared our data with other non-communicable diseases as a reference. According to the WHO disease and injury regional estimates for 2004 [34], the DALYs/1,000 due to CMS in our study were lower than those attributable to neuropsychiatric conditions in China’s 15- to 44-year-old male population, which caused 40.66 DALYs/1,000. However, the CMS DALYs/1,000 were higher than those for any other type of non-communicable condition. Unfortunately, a comparison between this work and the GBD study of non-communicable conditions is only partly possible because of differences in the data collection strategies and in the populations studied. The sample in the present study did not represent an entire nation or region, but rather was a specific immigrant population with high susceptibility to CMS. Nevertheless, the burden of CMS in the immigrant population may be considered to be high because approximately 3 months of healthy life is lost per CMS patient. This loss equates to approximately 2 weeks per immigrant when considered in relation to the population as a whole.

In previous reports, CMS has been found to affect 5-15% of the population at and above 3,200 m in Andean countries [35]. In a 4,300-m area in Peru, the prevalence was 6.8-15.4% in a miner population between the ages of 20 and 39. In Tibet, an overall prevalence of 5.6% in immigrant civilians was reported; in Lhasa immigrant civilians, the CMS rate was 2.2-8.7% [17]. However, despite some differences in diagnostic criteria, the results of the different studies are partially comparable because of the variance in environment, population characteristics, and geographical factors. In a similar young soldier population in Tibet, the CMS prevalence was reported to range from 9.3% (in Lhasa at 3,650 m) to 30.4% (in an area at 5,000 m) [36], which is similar to our findings (2.1-37.4%), although, our figures were lower for lower-altitude areas at 3,500-3,999 m above sea level (2.1-3.2%). The main reason for this discrepancy may be that their criteria were slightly lower (Hb > 200 g/L and SaO_2_ < 85%). In another report from Peru, in which the criteria were Hb > 213 g/L and SaO_2_ < 83%, a male population living at 4,300 m had a CMS prevalence of 15.6% [37], which is similar to our findings (4,000-4,499 m: 13.8-14.8%).

In this study, the prevalence of CMS significantly increased with ascent. When the altitude increased from approximately 3,500 m to 5,500 m, the prevalence increased approximately ten fold. Similarly, both the individual DALYs and the DALYs/1,000 also increased significantly with altitude. This result indicates that higher altitude not only increases the prevalence of CMS but also contributes to an aggravated CMS disease burden. For example, in the 3,500-3,999 m area for the total immigrant population, every immigrant lost nearly one-and-a-half days of healthy life annually because of CMS. In contrast, in the 5,000-5,400 m area, they lost one-and-a-half months of healthy life each year. When considering only the CMS patient, in the 3,500-3,999 m area, every CMS patient lost nearly two months of healthy life annually; in the 5,000-5,400-m area, they lost nearly three-and-a-half months of healthy life annually.

The statistical analyses indicated that some factors, including age, HSYs, BP, HR, smoking, and occupation, not only significantly differed between the CMS and non-CMS groups but also associated with the individual disease burden in the CMS population. The CMS population had a higher age, HSYs, BP, HR, smoking rate, and proportion of E&C occupations. These factors will now be discussed in relation to previous studies.

The study from Peru suggested that CMS is a clinical manifestation of aging at high altitudes in native highlands [38]. Other studies have also reported that high-altitude dwellers show earlier cardiovascular degenerative changes with aging [39,40]. The results of the present study indicate that the CMS group was older than the non-CMS group and had a longer highland service history. Age and HSYs were also positively associated with the individual DALYs of the CMS patients. In the sample population, the men always joined the army at the ages of 17–19 and were sent to the highland for service after 0.5 to 1 year of training; therefore, subjects of the same age had similar highland service years. Thus, the effects of age and HSYs cannot be discussed independently because both variables represent aging in the highland. Aging in the highland causes not only a greater number of CMS patients but also a heavier CMS disease burden, i.e., prolonged time spent in the highland could cause a healthy person to develop CMS [17] and could aggravate the severity of CMS symptoms [7]. Thus, redeploying soldiers seriously affected by CMS to a lower-altitude service area would help to decrease the health lost for the highland service population.

A previous study found that subjects with CMS showed reductions in the response to the stimulation of the peripheral chemoreflexes and in baroreflex control of heart rate and blood pressure, which correlated with increases in CMS scores and in hemoglobin levels [41]. It is also known that vasodilator action of hypoxia at the microcapillary level may be incapable of decreasing systemic hypertension [42]. In this study, we found that, compared with the non-CMS group, members of the CMS group had higher BP (both SBP and DBP) and HR; this result is consistent with the findings of other reports [43-45]. We also found that BP (especially DBP) and HR were also significantly positively associated with individual DALYs in CMS patients. This result implies a progressive impairment of cardiovascular regulation among the CMS patients. Accordingly, improving cardiovascular regulation may help to alleviate the disease burden of CMS.

As some scholars have suggested, smoking is a risk factor for altitude disease [9,46]. Previous research in the male Han population has suggested that the prevalence of CMS is three times higher in smokers than in non-smokers [9]. In the present study, the CMS group had a higher smoking rate than the non-CMS group. Moreover, smoking was also associated with higher individual DALYs in the CMS patients. The mechanism causing this effect could be that cigarette smoking worsens hypoxia, produces a lower oxygen-carrying capacity, causes centrilobular emphysema, reduces alveolar ventilation, and thus impairs cardiopulmonary function. Accordingly, smoking control among the highland immigrant population would decrease both the prevalence and the disease burden of CMS; the authorities should consider this possibility.

Patients with E&C occupations were more likely to develop CMS and had heavier disease burdens. The possible reason for this result is that these occupations are associated with a relatively heavy physical burden. Physical exertion leads to greater oxygen consumption, which aggravates hypoxia [47,48]. These occupations may also be more likely to bring people into contact with dust, waste gas, and cold weather, which potentially increase the risk of CMS. Thus, immigrants with E&C occupations in the highland should have more protection for CMS prevention and more compensation for health damages.

Regarding symptom categories, the greatest BOD due to CMS is associated with headaches, sleep disturbances, and breathlessness/palpitations, which made up almost three fourths of the total BOD. The high contribution of these symptoms to BOD is due to both their high prevalence and their high DW. These three symptoms may play an important role in the course of CMS, and they should be considered in measures to improve the quality of life for immigrant highlanders.

### Strengths and limitations

There are several strengths of this study. First, the validity of the CMS data obtained from the military medical surveys is likely to be high. The data were collected by trained investigators using consistent criteria; the investigators were able to adequately assess the young men’s health status in the vast majority of the cases. Second, this study improves on some earlier studies in which the comorbidity of DWs for different health states was determined by simple addition; this procedure leads to the DW exceeding 1 at the individual level. Comorbidity was handled in the DW and DALYs calculations in our work, which makes the results more reliable. Third, this study described the BOD based on data from recent years; therefore, the DALYs may represent the actual disease burden in the population. We attempted to improve the DALYs measure’s sensitivity by comparing two sequential years; the results of the two data sets revealed no significant differences. A further strength of this study is that the authors have made modest and sensible health improvement recommendations relating to the specific symptoms that were identified as problematic, thus avoiding the criticism made of many studies of this general type that, for a variety of reasons, the measures used are not suitable for use as a basis for resource allocation decisions [49].

However, the results of this study should be interpreted with caution for the following reasons.

First, in the study of DW, cultural differences may affect the determination of PTO values, and different cultural backgrounds might account for variations in the DW ratings. Because of the relative localization of the expert panel, the DWs derived from this study are limited and should be considered regional DWs. However, because our primary aim was to focus on Tibet and because most highland immigrants reside in this territory, the cultural difference problem may not have a great impact on this study.

Second, The PTO method has been questioned for having a lower test-retest reliability than the time-trade-off technique, which is another common technique used in DALYs studies [50]; however, this may be less of a problem when using the Delphi method.

Third, a case-based approach may cause a substantial increase in the amount of data required, whereas an integrated health registration system has not been used for the entire immigrant population; therefore, the authors selected a population of young servicemen as a sample. Consequently, the data were collected from a specialized male population in which age, diet, and behaviors were similar; this specialized population may limit the generalizability of the results to other populations.

Fourth, this study handled DALYs in the same way that we handled YLD. However, whether some people may have died from CMS is difficult to determine. Because of the confounding effects of other diseases and the difficulty attributing each case to CMS, some potential symptoms not listed in the Qinghai-CMS-questionnaire may have not been considered.

Moreover, the assumption that the duration of CMS is an entire year and the averaging of its severity over the course of a year are not precise; some symptoms may occur intermittently, and the severity may change over time. Nevertheless, a disease that is chronic in nature may not change too much on average over a year, and the purpose of this study was to investigate the population level, not individual level, CMS burden, therefore this assumption was acceptable in this study.

Finally, BOD studies can detect disease-specific and overall trends in BOD within a region or in a population over time [26]. This study, however, did not analyze the tendency of CMS to vary within an area; we investigated the BOD only within two neighborhoods in two recent years using a simple cross-sectional investigation. Thus, future studies may be necessary.

## Conclusion

CMS is one of the most important diseases that burdens the highlanders. Accordingly, CMS is a public health issue in Tibet. This study is the first step in evaluating the BOD in the highland areas to suggest the importance of BOD indicators when considering health compensation policies and workforce rotation strategies in the highlands. The results show that CMS imposes a considerable burden on young male highland immigrants. Immigrants with characteristics such as higher residential altitude, more advanced age, longer HSYs smoking behavior, and engineering or construction occupations were more likely to develop CMS and to increase the burden of disease. Higher BP and HR as a result of CMS were also positively associated with the disease burden, which implies a progressive impairment of cardiovascular regulation. Thus, the authorities should pay more attention and should create reasonable strategies to alleviate the disease burden of CMS. As a useful tool in high-altitude public health research, further development and application of a DALYs measure that focuses on the full disease spectrum in the highland population should be supported.

## Abbreviations

BOD: Burden of disease; BP: Blood pressure; CMS: Chronic mountain sickness; CV: Coefficient of variation; DALY: Disability adjusted life year; DBP: Diastolic blood pressure; DW: Disability weight; E&C: Engineering and construction; Hb: Hemoglobin; HSY: Highland service year; ICD-10: International Classification of Disease, Injuries, and Causes of death--tenth revision; SBP: Systolic blood pressure; SD: Standard deviation; WHO: World Health Organization; YLD: Years lost due to disability; YLL: Years of life lost; 95%CI: 95% confidential interval.

## Competing interests

The authors declare that they have no competing interests.

## Authors’ contributions

XL, TP, and YG conceived this study. TP, XL, FT and LZ conducted the Delphi process and performed calculation of DALY. TP, XL, HY, FT, and YL participated in the data collection and the redaction. FT and YL gave the material support. HX gave statistical support. XL wrote the first draft. TP and YG revised the manuscript. All authors read and approved the final manuscript. XL undertook the revision of manuscript.

## Pre-publication history

The pre-publication history for this paper can be accessed here:

http://www.biomedcentral.com/1471-2458/12/401/prepub
